# Molecular Characterization and Functional Regulation of Melanocortin 2 Receptor (MC2R) in the Sea Bass. A Putative Role in the Adaptation to Stress

**DOI:** 10.1371/journal.pone.0065450

**Published:** 2013-05-27

**Authors:** Maria Josep Agulleiro, Elisa Sánchez, Esther Leal, Raúl Cortés, Begoña Fernández-Durán, Raúl Guillot, Perry Davis, Robert M. Dores, Nicole Gallo-Payet, José Miguel Cerdá-Reverter

**Affiliations:** 1 Department of Fish Physiology and Biotechnology, Instituto de Acuicultura de Torre de la Sal, Consejo Superior de Investigaciones Científicas (IATS-CSIC), Castellón, Spain; 2 Department of Biological Sciences, University of Denver, Denver, Colorado, United States of America; 3 Department of Medicine, University of Sherbrooke, Sherbrooke, Québec, Canada; Centre of Marine Sciences & University of Algarve, Portugal

## Abstract

The activation of melanocortin 2 receptor (MC2R) by ACTH mediates the signaling cascade leading to steroid synthesis in the interrenal tissue (analogous to the adrenal cortex in mammals) of fish. However, little is known about the functional regulation of this receptor in fish. In this work described, we cloned sea bass MC2R from a liver cDNA. SbMC2R requires the melanocortin 2 receptor accessory protein (MRAP) for its functional expression. Dietary cortisol but not long-term stress protocols downregulated interrenal sbMC2R expression. Data suggest the existence of a negative feedback on interrenal sbMC2R expression imposed by local or systemic glucocorticoids. This feedback could be involved in long-term stress adaptation by regulating interrenal sensitivity to ACTH. ACTH-induced MC2R activation stimulates hepatic lipolysis, suggesting that ACTH may mediate stress-induced effects upstream of cortisol release.

## Introduction

Melanocortin peptides are processed from the complex peptide precursor named proopiomelanocortin (POMC). They are mainly comprised of adrenocorticotropic hormone (ACTH) and melanocyte-stimulating hormones (α-, β- γ- and δ-MSH). POMC is mainly produced in the pituitary and its posttranslational processing occurs in a tissue-specific manner [1]. The proteolytic cleavage of POMC by prohormone convertase 1 (PC1) generates ACTH and β-lipotropin in the corticotrophs of the anterior pituitary, whereas cleavage by PC1 and PC2 produces α-MSH and β-endorphin in the melanotrophs of the pars intermedia [2]. The melanocortins exert its physiological role by binding to a family of specific G protein-coupled receptors (GPCR) that positively couple to adenylyl cyclase. Tetrapod species have five melanocortin receptors (MC1R-MC5R), although the number of receptors diverges in teleost fish. For example, pufferfish have only 4 receptors with no melanocortin MC3R, while zebrafish (*Danio rerio*) has six MCRs, with MC3R and two copies of the melanocortin MC5R [3].

The MC2R is specifically activated by ACTH, while the other MCRs can be activated by the MSHs as well as ACTH [4]. Cell surface expression of a functional MC2R strictly requires the presence of a single-transmembrane domain accessory protein called the MC2R accessory protein (MRAP). This protein interacts with the receptor to facilitate correct folding, subsequent glycosylation and cell surface expression [5] but it is also essential for ACTH binding and ACTH-induced cAMP production [6]–[8]. A recent study indicates that MC2R is present as a homodimer in the plasma membrane. This homodimer oligodimerizes with two MRAP homodimers to form a hexameric complex [9]. In the absence of MRAP, the MC2R is retained at the endoplasmic reticulum where cells cannot be stimulated by ACTH. In mammals, the alternative splicing of the last two exons of MRAP gene gives rise to two isoforms that differ in the C-terminal region (MRAPα and MRAPβ; [5]) but MRAP isoforms were not found in zebrafish [8]. In the latter species, MRAP and MC2R are mainly expressed within the interrenal (analogous to the adrenal cortex in tetrapods) and hepatic tissue, where they play a key role in interrenal stereoideogenesis and probably the hepatic lipid metabolism [8]. An additional paralogue of MRAP known as MRAP2 is also expressed in vertebrate species, including fish [3]. Because of the finding of MRAP2, the original protein responsible for the traffic of the MC2R to the plasma membrane should be called MRAP1 instead of MRAP.

As in other vertebrates, MC2R activation also plays an essential role in the stress response in fish. Following stressor exposure, the hypothalamic neurons release corticotrophin-releasing factor (CRF) to the anterior pituitary, which stimulates ACTH production and release from corticotrophs. With the permission of MRAP1, plasma ACTH activates interrenal MC2R, which results in the increased synthesis of cortisol, the main glucocorticoid in fish. Cortisol is released to the blood to regulate a wide array of systems in both stressed and non-stressed animals [10]–[12]. Although *in vitro* and *in vivo* ACTH-induced interrenal steroideogenesis [10] is well established, few studies has focused on the MC2R function and its characterization in fish [8], [13]–[15]. Our previous work demonstrated that sea bass (*Dicentrarchus labrax*) is very sensitive to stressful conditions [16], which induce a severe decline in food intake levels and growth performance [17]. To better understand this sea bass response to stressful conditions, we cloned MC2R (sbMC2R) and studied the liver and interrenal response to metabolic and chronic physical stress, respectively, as well as the cortisol-induced effects on MC2R interrenal expression.

## Results

### Molecular cloning sbMC2R

**Figure 1:**
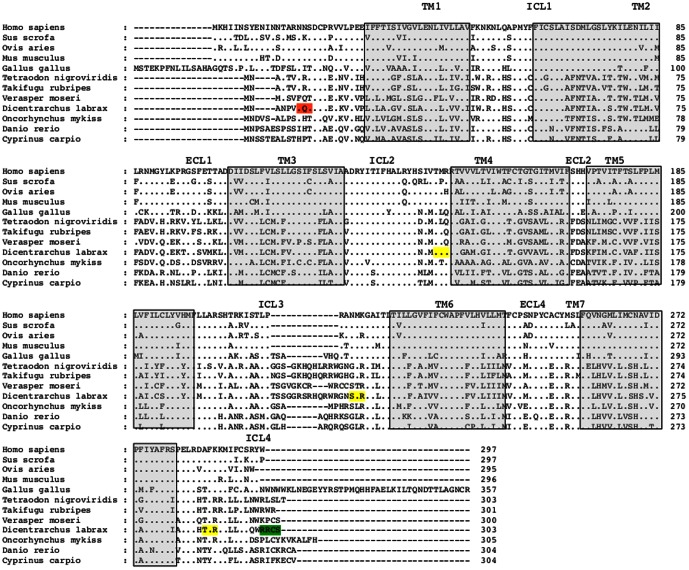
Alignment of MC2R amino acid sequences. Sea bass MC2R sequence is highlighted in bold letters. Dots indicate amino acids identical to the top sequence. Dashes were introduced to improve alignment. Grey boxes show putative transmembrane domains. Putative N-linked glycosylation sites are coloured orange. Blue indicates potential casein kinase II phosphorylation sites. Putative protein kinase C phosphorylation sites are coloured yellow. Green color frames show cAMP- and cGMP-dependent protein kinase phosphorylation sites. Sea bass MC2R sequence accession number FR870225

By means of RT-PCR and using degenerate primers designed against conserved regions of fish melanocortin receptor sequences, we cloned a 214 bp fragment showing high identity to the MC2Rs reported in other vertebrate species. The full cDNA sequence was obtained by screening a liver cDNA library with the fragment obtained by RT-PCR. The excised fragment contained an open reading frame of 909 bp that encodes a protein of 303 amino acid residues length protein with seven potential transmembrane domains. Similar to other melanocortin receptors, the sbMC2R orthologue exhibits short extracellular (ECL) and intracellular (ICL) loops, and shares cysteine residues at positions 234, 248 and 254 (sea bass numbering), which are fully conserved in all melanocortin receptors (Fig. 1 and http://www.gpcr.org). The deduced amino acid sequence displays a potential N-glycosylation site within the N-terminal domain at position 7 (Fig. 1). Sea bass MC2R shares the PMY motif in the first ICLD, which is conserved in most melanocortin receptors. The motif DRY in the ICL2, a consensus motif of the class A of rodopshin-like G-protein coupled receptor, is also present. Consensus recognition sites for protein kinase C (PKC) were found at positions 133 (ICL2), 217 (ICL3) and 289 in the extended intracellular tail (ICL4). Similarly, one cAMP- and cGMP-dependent protein kinases and two casein kinase II were found at positions 300 (extended intracellular tail), 28 (TM1) and 272 (TM7), respectively. The presence of consensus sites for diverse kinases suggests regulation of the receptor by phosporylation. The identity of the deduced amino acid sequence ranged from 55–79% compared with other fish MC2R sequences, but was less than 44% compared with the tetrapod MC2R sequences. The identity was also unequally distributed. The average identity for the different segments of the receptor was calculated following the alignment shown in Figure 1. The average identity for the different TMs was 54.1% (TM1), 62.2% (TM2), 68.6% (TM3), 48.4% (TM4), 48.9% (TM5), 58.5% (TM6) and 65.6% (TM7). The highest identity values were found within ICL1 (73%), ICL2 (82.2%) and ECL4 (77.4%).

### Peripheral and central distribution of sbMC2R mRNA

**Figure 2:**
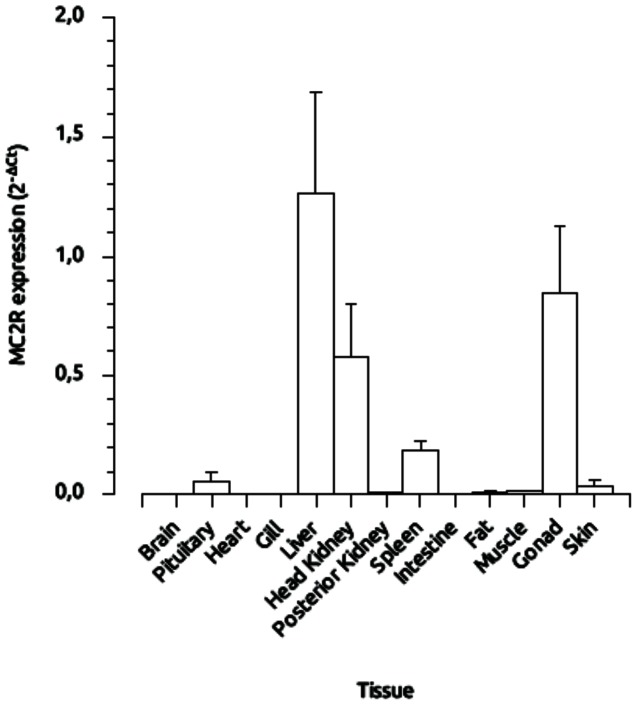
Distribution of sea bass MCR2 mRNA expression in different tissues, as revealed by q-PCR. β-actin was used as reference gene. See material and methods for details.

Higher sbMC2R mRNA levels were detected by real time PCR in the liver, testis and head-kidney but expression level was also detected in the pituitary and spleen. Low levels were distinguished in the fat, muscle and skin (Fig. 2).

### SbMC2R activation by ACTH

**Figure 3:**
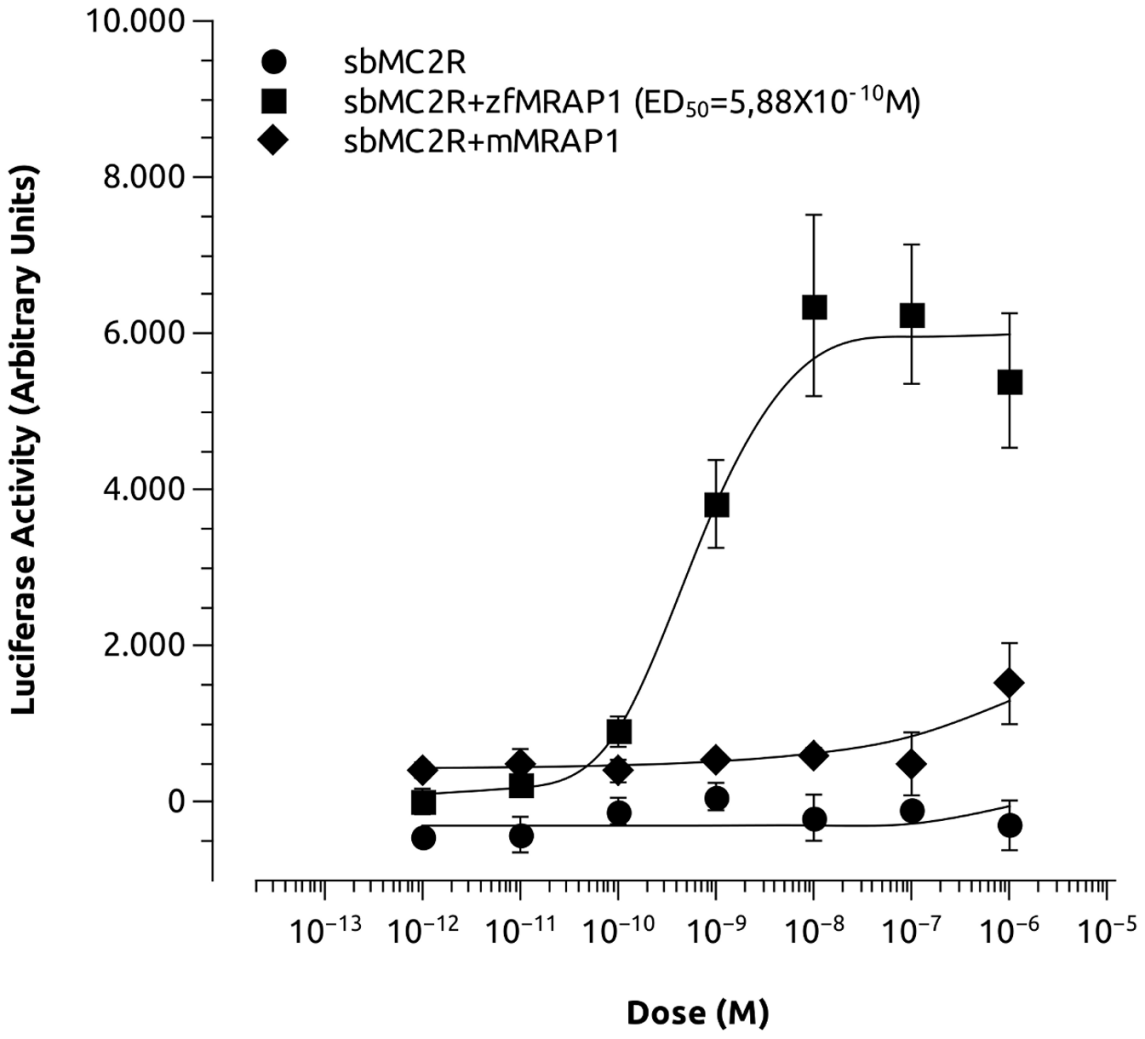
Effects of human ACTH (1–24) on luciferase activity in CHO cells transiently expressing sbMC2R, and zebrafish (zf)MRAP1 or mouse (m)MRAP1. Cells were transiently transfected also with a cAMP-responsive luciferase reporter gene.

For functional characterization of the sbMC2R, the coding region was directionally ligated into pcDNA3 and transiently expressed in CHO cells also producing luciferase under the control of cAMP responsive elements. The functional expression of MC2R requires the presence MRAP1 which was co-transfected with the sea bass receptor. Since sea bass MRAPs have not been characterized, we used mammalian (mouse) and zebrafish MRAP1. The stimulation of sbMC2R with hACTH(1–24) in the absence of MRAP1s did not induce any change in the luciferase activity. However, when zfMRAP1 was co-transfected the sbMC2R-induced luciferase activity increased in a dose response manner (ED_50_ = 5,88×10^−10^) (Fig. 3). When co-expressed with mMRAP1, the sbMC2R was able to respond only to the highest hACTH(1–24) doses (10^−6^ M).

### ACTH effects on hepatic lipid metabolism

**Figure 4:**
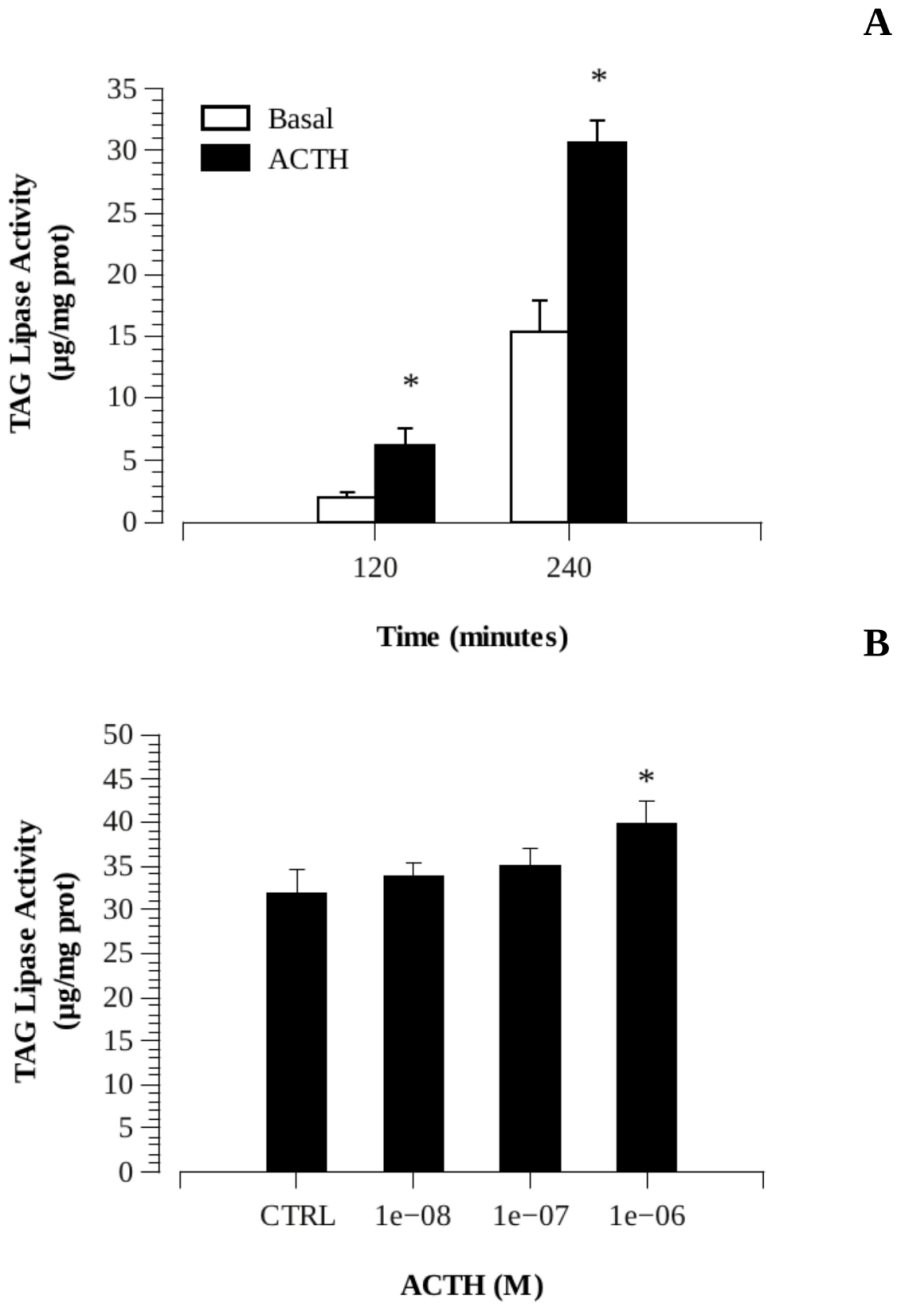
Effects of melanocortin agonist ACTH on hepatic lipolysis measured as releasing of non-esterified fatty acids (NEFA) after hormonal treatment to the culture media. A) Time course experiments using human ACTH at 10^−6^ M after 2 and 4 hours incubation. B) Dose-response experiments after 4 h incubation.

The incubation of liver slides with hACTH 10^−6^ M in HBS resulted in a significant increase in total NEFA concentration in the culture medium after 2 and 4 h (Fig. 4A). Only the highest hACTH concentration significantly stimulated NEFA production *in vitro* (Fig. 4B).

### Effects of fasting and stress on sbMC2R expression

**Figure 5:**
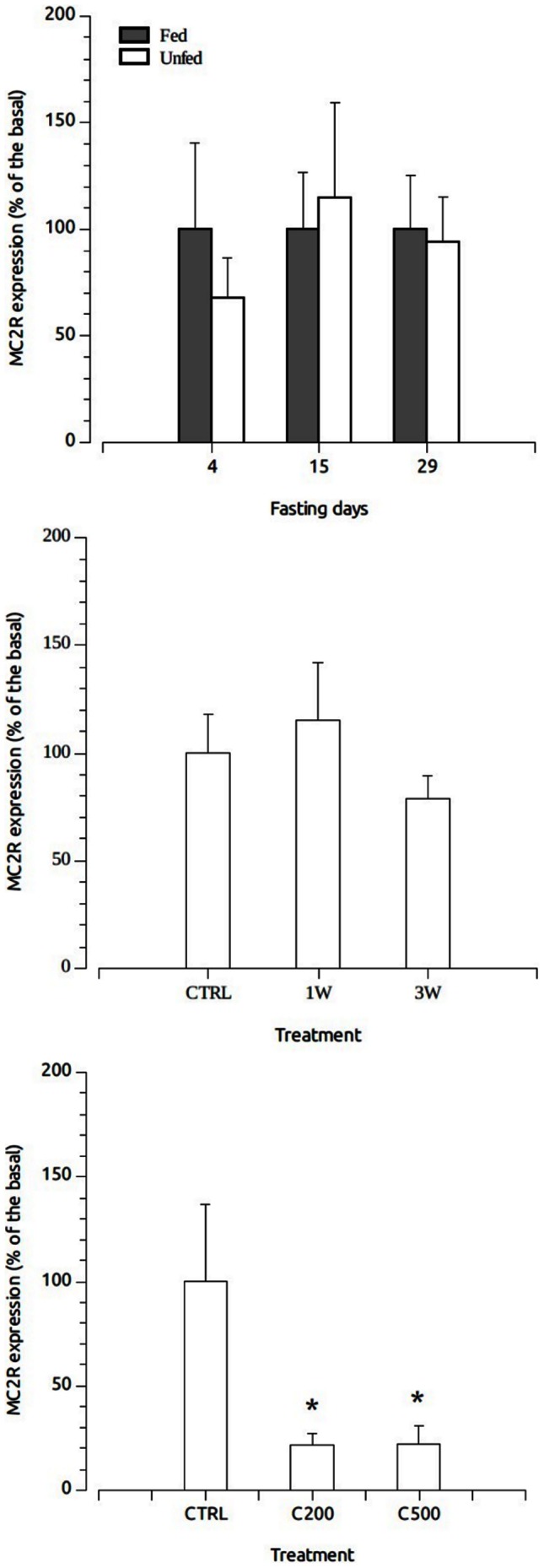
Physiological and hormonal regulation of MC2R expression. A) Effects of progressive fasting on liver MC2R expression. Data were expressed as percentage of the control level. B) MC2R expression levels in interrenal tissue of the sea bass after repetitive physical disturbance imposed by routine cleaning protocols with animals inside the tanks once (1W) or three times a week (3W) during 33 days. Control fish (CTRL) were never disturbed C) Interrenal MC2R expression levels in sea bass fed with cortisol-containing diets at doses of 0 (CTRL), 200 (C200) and 500 (C500) µg/g food. Animals were fed during 32 days using self-feeding systems. Asterisk indicates significant differences between control and treated fish after ANOVA followed by Tukey's multiple range test (*P*<0.05). Gene expression levels were expressed as ratio specific mRNA/β-actin. Similar results were obtained when the expression levels were expressed as ratio specific mRNA/18S RNA. Data are mean ± SEM (n = 8).

Quantitative PCR yielded no significant differences in hepatic MC2R-mRNA expression levels when fed and fasted animals were compared after 4, 15 and 29 days of fasting (Fig. 5A). Similarly, repetitive physical stressors induced no significant differences in the interrenal expression of the receptor (Fig. 5B). In contrast, long-term administration of cortisol-containing diets induced a significant decrease in interrenal sbMC2R expression (Fig. 5C).

## Discussion

The present study demonstrates that sbMC2R requires the presence of MRAP1 to reach its functional expression. In the interrenal tissue, dietary cortisol decreased MC2R expression but chronic physical stressors had no effect. In addition, the receptor was profusely expressed in the sea bass liver where it stimulated lipolysis. Together, these data suggest that MC2R/MRAP1 activation by ACTH is essential in the regulation of fish stress response. Such a response could be partially orchestrated upstream of the concomitant ACTH-induced cortisol release. However, the regulation of MC2R by dietary cortisol suggests the existence of a short-loop feedback that could play an important role in the adaptation to stress.

Sequence comparisons show that the cloned receptor in the sea bass displays high identity to other vertebrate MC2R. Sequence analysis reveals that MC2Rs have a very short N-terminal region. In the sea bass, this region expands only the first 19 amino acids and exhibits one potential glycosylation site which is conserved in all MC2R molecules. The N-terminal region is critical in receptor trafficking since it partially determines the presence of the receptor in the plasma membrane [18], [19]. The MC2R is intracellular arrested when expressed in non-adrenal cells and MRAP1 expression allows its cell surface functional expression [5]. The replacement of the MC2R N-terminal region by that from MC4R permits the surface expression, albeit inefficiently, demonstrating that the N-terminal region plays a key role in the intracellular retention of the receptor [18]. N-linked glycans are also required for plasma membrane targeting of MC2R. The absence of N-glycosylation in the human MC2R blocks the surface expression of the receptor [19]. It has been proposed that the low N-glycosylation level of MC2R could constrain the ability of the receptor to reach the cell membrane. Oligomerization with two antiparallel MRAP1 molecules [9], [20] could increase the N-glycosylation level of the MC2R-MRAP1 complex, since MRAP1 exhibits two sites for N-linked glycosylation, thus facilitating surface expression [18], [21], [22].

Pharmacological experiments demonstrated that sbMC2R requires the presence of MRAP1 for its functional expression in CHO cells. In the absence of MRAP1, sbMC2R did not respond to hACTH(1–24) stimulation, whereas co-expression of mammalian MRAP1 in the cell system allowed only a very discrete receptor response. Therefore, the MRAP1 dependence of MC2R could be an ancestral requirement of this receptor but recent experiments with elephant shark (*Callorhinchus milii*) have reported that elasmobranch MC2R responds to ACTH independently of MRAP1 [22]. We have previously demonstrated that zfMC2R requires zfMRAP1 coexpression to traffic to the plasma membrane and to generate a signal in response to ACTH [8]. Here, we further shown that mammalian MRAP1 cannot interact efficiently with sbMC2R, to allow an intracellular signal in response to hACTH(1–24). Similar results were obtained also in rainbow trout (*Onchorynchus mykiss*) [24]. In some species, such as tilapia (*Oreochromis mossambicus*) or barfin flounder, (*Verasper moseri*), MC2R was shown to be activated by different isoforms of MSH by using interrenal primary cell cultures but interrenal MC5R expression was also reported [25], [26]. We did not test the effect of MSHs on sbMC2R activity but our previous studies in zebrafish demonstrated that α-MSH was not able to stimulate the receptor [8]. Similar results were also obtained in trout and carp by using primary cultures and measuring the cortisol released to the culture medium [14], [15].

In mammals, MC2R is predominantly expressed in the adrenal cortex, where it regulates adrenal steroid synthesis and secretion. In teleosts, the steroidogenic cells, together with closely intermingled chromaffin cells, are embedded in the head kidney, forming the interrenal organ, homologue of the mammalian adrenal gland [27].We show that sbMC2R is densely expressed in the head kidney but also in liver, spleen, and testis. In addition, some level of expression was detected in the pituitary. A similar distribution was reported in trout, carp, zebrafish and flatfish [8], [14], [15], [26]. Both head-kidney and spleen contain a high number of macrophages [28], suggesting that MC2R activation could be involved in the modulation of the sea bass immune system, as previously proposed [29]. Microarray studies have demonstrated the effects of handling stress on the expression of immune system-related genes in fish [30]. In addition, ACTH has been shown to modulate cytokine expression in the gilthead seabream (*Sparus autara*, [31]). Therefore, ACTH-MC2R-MRAP1 interaction in the immune tissue could represent an endocrine pathway that regulates the effect of stress on the immune response upstream of cortisol release by interrenal tissue.

In our experimental conditions, the chronic physical stressors could not regulate interrenal sbMC2R expression but when cortisol was added to the diet daily, a significant reduction in the interrenal expression levels was consistently detected. Experiments in dogs have demonstrated that the abundance of the mRNA encoding MC2R is significantly downregulated in cortisol-secreting adrenocortical carcinomas [32]. This suggests that cortisol regulates interrenal expression of the receptor and by extension the sensitivity to ACTH in a negative short-loop feedback. *In vitro* experiments in rainbow trout have demonstrated that ACTH can upregulate interrenal receptor expression [15]. Therefore, ACTH could increase its own interrenal responsiveness but cortisol, synthesized and secreted in response to the systemic ACTH, would modulate negatively the system to reach the original receptor levels prior to stimulation. This regulatory system could take part in the stress adaptation of the HPI axis. The existence of a negative intra-adrenal feedback loop has previously been suggested in mammals [33]. Studies in carp have shown that restraint stress for 1 day did not induce changes in the interrenal MC2R expression but a significant reduction was seen after 7 days of treatment [14]. Results obtained in trout also demonstrated an acute-stressor-induced elevation of plasma ACTH and interrenal MC2R expression [15] 4 hours post-stressor. In our experiment, sea bass were physically disturbed once or three times a week for 33 days. At the end of the experiment, the plasma cortisol levels were monitored and no differences were found. However, previous pilot experiments demonstrated that this stress protocol induced a significant increase in plasma cortisol after 2 hours [17]. It is therefore plausible that during chronic stress, sea bass cannot maintain high corticosteroid levels, because of metabolic implications. These high cortisol levels could counteract ACTH-induced MC2R autoregulation, leading to a normalization of interrenal receptor levels, ACTH responsiveness and, by extension, to the normalization of cortisol levels. Previous studies in the sea bass showed that short-term confinement (4 h) did not induce a loss of interrenal sensitivity to ACTH [34]. However, long-term crowding (23 days) in gilthead seabream reduced the sensitivity of interrenal tissue to ACTH stimulation [35]. This hypothesis would support the absence of differences in cortisol plasma levels and MC2R mRNA during long-term stress protocols in the sea bass [17].

There are no studies reporting the functional involvement of MC2R in the brain of vertebrates. In mammals and avian species, MC2R does not seem to be expressed in the brain [36]-[38] but several studies in fish, including rainbow trout [15], Fugu (*Takifugu rubripes*, [13]) and barfin flounder, (*Verasper moseri*, [26]), have demonstrated that this receptor is profusely expressed in the central nervous system. However, studies in carp (*Cyprinus carpio*, [14]) and zebrafish [21] failed to show central MC2R expression. The expression of MC2R in the fish brain is intriguing and it could be relevant in the regulation of the central response to stressors upstream of cortisol release. However, more studies involving *in situ* hybridization and or ACTH binding are required.

In the gonadal tissue, sbMC2R was expressed in the testis and residual levels were found in the ovary (not shown). Experiments in rat have demonstrated that ACTH stimulates testosterone production in neonates, while adult Leydig cells were insensitive to melanocortin peptides [39]. Interestingly, gonadotropin-independent precocious puberty has been reported in boys with a congenital mutation in *DAX 1* that results in adrenocrotical hypoplasia, increased testosterone and ACTH levels [40]. Although stress effects of fish reproduction are documented [41], to the best of our knowledge, no studies have focused on the effects of ACTH on the fish male reproductive axis. However, studies in zebrafish have reported the involvement of ACTH in gonadotropin-stimulated estradiol release form ovarian follicles [42]. Altogether, MC2R expression in the sea bass testis suggests that ACTH could be involved in the regulation of the testicular function and, particularly, in the regulation of the stereoideogenic pathways.

In a previous work, we demonstrated the effect of MTII (melanotan II) on the hepatic lipid metabolism of the sea bass [43], an effect probably mediated by the expression of MC5R in the liver. Following the same protocol, we now demonstrate that ACTH can also induce hepatic lipolysis, measured as free fatty acid release into the culture medium after melanocortin agonist exposure of liver fragments. The lipolytic effects were only detected when the highest doses of hACTH (10^−6^ M) were used. It contrasts with the high sensitivity of the receptor by ACTH measured by in vitro expression of the MC2R in CHO cells. This discrepancy can be attributable to the different culture systems (monolayer cell culture *vs* tissue fragments), MC2R expression levels and/or evaluated response (luciferase activity *vs* lypolysis). It is well known that stress activation of the hypothalamus-pituitary-interrenal (HPI) axis contributes in a key way to restoring energy homeostasis by mobilizing fuel stores to make energy available for increased metabolic demand [44]. Chronic stress induces a severe decline of the hepatosomatic and mesenteric fatty index in the sea bass as well as in the food intake levels and growth. However, no differences in plasma cortisol levels were detected [17]. It is thus conceivable that ACTH promotes hepatic lipolysis independently of cortisol release. We thought that fasting might regulate hepatic sensitivity to ACTH by modulating receptor expression levels, but long-term fasting had no effect on hepatic sbMC2R expression.

In summary, the sbMC2R orthologue was cloned form a liver cDNA library. Comparative analysis of MC2R sequences suggests that ICL2 and ICL3 are key areas of receptor activation, whereas ECL4 could be involved in agonist binding. Functional expression in non-adrenal cells requires the presence of MRAP1. Interrenal expression was modulated by dietary cortisol, suggesting the existence of a negative feedback on MC2R expression imposed by local or systemic glucocorticoids. This feedback could be involved in long-term stress adaptation by regulating interrenal sensitivity to ACTH via the modulation of MC2R expression, resulting in normalized plasma glucocorticoid levels. The fact that ACTH-induced MC2R activation stimulates hepatic lipolysis suggests that ACTH mediates stress-induced effects upstream of cortisol release.

## Materials and Methods

### Animals and reagents

One year old immature sea bass were maintained in 2000 L tanks supplied with continuously aerated running sea water and equipped with an automatic feeder activated by a string sensor placed 3 cm below the water surface. The feeders were connected to a computer system that recorded the date, the time and the tank from which each food demand originated. The number of demands was integrated every 5 minutes. Animals were maintained under natural conditions for at least six months and self-fed with a commercial diet (Mistral 21, Proaqua Nutrición, S.A.; 43% protein, 23% fat, 20% carbohydrates, 6% ash, gross energy 22.5 kJ/g, in 3 mm standard pellets). Before the experiments, fish were placed in experimental 500 L tanks, continuously supplied with running seawater and provided with identical self-feeding systems, and acclimated for at least one week. Prior to netting, animals were pre-anaesthetized in 2-phenoxy-ethanol (0.005%) for 3–5 minutes in their home tanks. Subsequently, the animals were removed from their home tanks and anesthetized for 2 min in the same anesthetic (0.05%) in the sampling tank. The day before samplings, sensors were removed from the water at 10.00 am, the time at which sampling always started. When required, the experimental animals were sacrificed by rapid decapitation. All experiments were carried out in accordance with the principles published in the European animal directive (86/609/EEC) for the protection of experimental animals and approved by the Consejo Superior de Investigaciones Científicas (CSIC) ethics committee (project number AGL2007-65744-C03-02). Unless otherwise indicated, all reagents were purchased from Sigma (St Louis MO, USA).

### Cloning procedure

Genomic DNA isolated from blood was used as template for touchdown PCR reactions with Taq DNA polymerase (Invitrogen) and degenerate primers designed against conserved regions of the known MC2R sequences. The 5′ primer (MCR2Fw2) was a 18-mer with the sequence: 5′ CCATGCCMRSARGATTGC 3′. The 3′ primer (MCR2Rv2) had the sequence 5′ TGYAGCTSRAASAKAGATCGGT 3′. PCR products of about 214 base pair (bp) were isolated from low melting point (LMP) Nusieve GTG agarose gel (FMC) ligated into pGEM-T easy vector (Promega) and subsequently transformed into XLI-Blue E. coli. One clone that contained an insert of the expected size was sequenced. Subsequently, filters from a sea bass liver ZAP cDNA library (kindly supported by Dr. Giovanni Bernardini from the Department of Biotechnology and Molecular Sciences of University of Insubria, Italy) containing approximately 5×10^5^ clones were screened with the previous sbMC2R fragment obtained by PCR (see above). Membranes were prehybridized for at least 3 hours in hybridization solution (50% formamide, 6X SSPE, 0.5% SDS, 5 X Denhardt's solution and 10 mg/ml yeast tRNA type III (Sigma, St Louis, MO), 1 X SSPE containing 150 mM NaCl, 1 mM EDTA, 9 mM NaH2PO4, pH = 7.4). The probe was labeled with dCTP [α-32P] (Amersham Biosciences), using the random primer labeling kit (Invitrogen). Hybridization was carried out overnight in fresh hybridization solution containing 10^6^ cpm/ml dCTP [α-32P] at 42°C. Final washes were performed in 0.1 X SSPE at 60°C. After three purification rounds, six positive phages were selected for *in vivo* excision. The DNA insert in excised pBluescript II SK vector was sequenced on both strands. The nucleotide sequence of sbMC2R has been deposited with EMBL Nucleotide Sequence Database under accession number FR870225.

### Real time quantitative PCRs

For tissue distribution of sbMC2R expression, total RNA was purified from fresh tissues (testis, intestine, fat, liver, muscle, spleen, head kidney, posterior kidney, gill, skin, heart, pituitary and brain) and treated with RQ1-DNAse (Promega). Five micrograms were retrotranscribed using superscript II reverse transcriptase (Invitrogen) and random hexaprimers (Invitrogen). To evaluate gene mRNA levels from individual liver or head kidney total RNA was treated with RQ1-DNAse. Subsequently, three microgram aliquots were used as template for cDNA synthesis, which was primed as before.

One microlitre of cDNA (sbMC2R) or diluted cDNA (18S RNA and β-Actin) and primers (70 nM) were added to 7.5 µl of Sybr green PCR master mix (ABgene, Thermo Scientific, Spain) and the volume was adjusted to 15 µl with water. Primer sequence were qPCR_MC2R_F1 (5′ TTGCAGTGGACCGTTACATC3') and qPCR_MC2R_R1 (5' GGCAACGAAGCAGATCATGA 3'). PCRs were carried out on an iCycler (Bio-Rad, Madrid, Spain). Data were analyzed with the ΔΔCt (cycle threshold) method. As internal controls fragments of the sea bass β-actin mRNA and 18S RNA weres amplified, using primers qPCR_β-actin_Forw/qPCR_β-actin_Rev and qPCR_18S_Forw/qPCR_18S_Rev primers, respectively. Sequences were as follows: qPCR_β-actin_Forw 5' GCCGCGACCTTACAGACTAC 3' qPCR_β-actin_Rev 5' AGCACAGTGTTGGCGTACAG 3', 18S_Forw 5′ GCATGCCGGAGTCTCGTT 3′ and 5′ 18S_Rev 5′ TGCATG GCCGTTCTTAGTTG 3′ [45]. All samples were processed in duplicate.

### Constructs and pharmacological experiments

The full coding region of the sbMC2R was amplified by PCR using the vector isolated from the library as template and the primers Hind-MC2R-Forwad primer (5' TATAAGCTTATGAATGCTAACCCAGTG 3') and XhoI-MC2R-Reverse (5' TTACTCGAGGTAAAGCACATATAAAGTGT 3'). The receptor was then directionally subcloned into pcDNA3 (Invitrogen) and sequenced on both strands. The synthesis of zfMRAP1, mMRAP1 and hMC2R constructs was described previously [24].

Experiments were done in transiently transfected CHO cells. CHO cells were grown in DMEM/F12 with 5% fetal calf serum at 37°C in a humidified 5% CO_2_ incubator. Cells were plated in 96-well dishes and transfected with 40 ng/well total DNAusing FugeneHD according to the specifications of the manufacturer. Typically transfections were done with 13,3 ng/well of a cDNA dependent luciferase reporter containing multiple copies of cAMP responsive element (CRE) from rat insulin promoter, 21,3 ng/well sbMC2R DNA and 5,3 ng/well MRAP1 or GFP DNA. After 24 hours the medium was replaced and 40 µl fresh media containing 20 µM forskolin or hACTH (1–24) (Phoenix Biochemicals). After 5 hours medium was removed and One step luciferase Step Reargent (Nanolight Technologies) was added. Luminiscence was read on a BioTek platereader. Values shown represent the mean SEM from triplicate wells in a representative experiment (see [23], [24] for more details).

### Effects of ACTH on hepatic lipolysis

To evaluate the effects of melanocortin agonist on hepatic lypolysis, the animals were sacrificed and their livers carefully removed. Small liver slices (50–80 mg) were dissected and incubated in 1 ml HB medium (136.9 mM NaCl, 5.4 mM KCl, 0.81 nM MgS0_4_, 0.44 mM KH_2_PO_4_, 0.33 mM Na_2_HPO_4_, 5 mM NaHCO_3_, pH = 7.6), containing 5 mM glucose, 1.5 mM CaCl_2_ and 2.5% fat-free BSA for 60 min at 25°C. Subsequently, the medium was removed and slices were incubated with 1 ml of HBS containing human ACTH 10^−6^ M (Bachem). After 2 and 4 h at 25°C the medium was removed for the determination of non-esterified fatty acids (NEFA) using commercial kits (WAKO Diagnostics) and following the supplier's recommendations. Liver slides were mechanically homogenized for protein determination using the BCA protein assay kit (Pierce). Subsequently, dose-response studies were made by incubating liver slides with hACTH in HB ranging from 10^−6^ to 10^−9^ M for 4 h. Experiments were always performed in quadruplicate wells and repeated at least three times independently.

### Effects of progressive fasting on liver sbMC2R expression

To evaluate the effects of fasting on liver sbMC2R expression, ten groups of 10 fish each [body weight (BW)  = 117±1.54 g] were adapted for one-week to individual 500-litre aquaria and fed *ad libitum* at 9.00 a.m. After this acclimation period, five groups were hand fed at the same ratio, and the other five were fasted. Fed and fasted groups were sampled at 12.00 (3 hours post-feeding in the case of the fed groups) at 4 and 15 and 29 days. Fish were decapitated and liver samples were dissected for immediate total RNA extraction. RNA samples were kept at −80°C in 75% ethanol until cDNA synthesis for quantitative PCRs (see above).

### Effects of cortisol and chronic physical stress on interrenal sbMC2R expression

A first trial was designed to evaluate the effects of chronic physical stress on interrenal sbMC2R expression. One hundred animals (BW = 221.82±1.32 g L = 25.95±0.047 cm) were distributed into 10 experimental tanks (n = 10) provided with automatic self-feeders. Three tanks were cleaned once a week (1W) and three tanks were cleaned three times a week (3W), whereas the four control tanks were never cleaned (CTRL). The cleaning protocol was always performed at 10.00 am and involved draining and brushing the tanks with the animals inside. The tanks were emptied until the dorsal fins of the fish were exposed and then brushed for 2 minutes and immediately refilled. After 33 days, nine animals/treatment were sampled to obtain interrenal tissue samples.

A second trial was designed to evaluate the effects of cortisol administration on sbMC2R expression. Ninety animals (BW = 136±0.96 g L = 22.63±0.05 cm) were distributed into 9 experimental tanks (n = 10) provided with automatic self-feeders. The fish of three tanks were fed the control diet (CTRL), three tanks the cortisol-contaning food at 200 µg/g food (C200) and the remaining three tanks with cortisol-enriched diet at 500 µg/g food (C500) for 32 days. The animals were sampled as above and tissue samples were kept at −80°C until used. For detailed experimental design see [17].

### Data analysis and statistics

Sequence comparisons and alignments were performed using ClustalX. A phylogenetic tree was derived using public domain CulstalX, which uses the Neighbor-Joining method on a matrix of distances. The membrane protein secondary structure was predicted using the Split 4.0 Server (http://split.pmfst.hr/split/4/). In gene expression studies, specific mRNA levels were normalized as a ratio to 18S RNA. Statistical analysis was conducted by one-way analysis of the variance followed by Tukey's multiple range test (p<0.05). Putative transmembrane domains were inferred by TMHMM Server v. 2.0 (http://www.cbs.dtu.dk/services/TMHMM/).
